# Impact of the COVID-19 Pandemic on Antimicrobial Consumption and Hospital-Acquired Candidemia and Multidrug-Resistant Bloodstream Infections

**DOI:** 10.3390/antibiotics9110816

**Published:** 2020-11-17

**Authors:** Ana Belen Guisado-Gil, Carmen Infante-Domínguez, Germán Peñalva, Julia Praena, Cristina Roca, María Dolores Navarro-Amuedo, Manuela Aguilar-Guisado, Nuria Espinosa-Aguilera, Manuel Poyato-Borrego, Nieves Romero-Rodríguez, Teresa Aldabó, Sonsoles Salto-Alejandre, Maite Ruiz-Pérez de Pipaón, José Antonio Lepe, Guillermo Martín-Gutiérrez, María Victoria Gil-Navarro, José Molina, Jerónimo Pachón, José Miguel Cisneros, On behalf of the PRIOAM Team

**Affiliations:** 1 Infectious Diseases Research Group, Clinical Unit of Infectious Diseases, Microbiology and Preventive Medicine, Institute of Biomedicine of Seville (IBiS), University of Seville/CSIC/University Hospital Virgen del Rocio, Avenue Manuel Siurot, 41013 Seville, Spain; anaguigil@gmail.com (A.B.G.-G.); carmeninfanted@gmail.com (C.I.-D.); german.penalva@gmail.com (G.P.); juliapraena@gmail.com (J.P.); cristinaroca85@gmail.com (C.R.); lola_navarro_amuedo@hotmail.com (M.D.N.-A.); maguilarguisado@yahoo.es (M.A.-G.); nurinea@hotmail.com (N.E.-A.); manu.poyato@gmail.com (M.P.-B.); s.saltoalejandre@gmail.com (S.S.-A.); maite.ruiz.sspa@juntadeandalucia.es (M.R.-P.d.P.); josealepe@gmail.com (J.A.L.); Guiller_MG86@hotmail.es (G.M.-G.); josemolinagb@gmail.com (J.M.); pachon@us.es (J.P.); 2 Clinical Unit of Pharmacy, University Hospital Virgen del Rocio, 41013 Seville, Spain; mariav.gil.sspa@juntadeandalucia.es; 3 Clinical Unit of Emergency, University Hospital Virgen del Rocio, 41013 Seville, Spain; 4 Clinical Unit of Cardiology, University Hospital Virgen del Rocio, 41013 Seville, Spain; marian.romero.sspa@juntadeandalucia.es; 5 Department of Medicine, University of Seville, 41004 Seville, Spain; 6 Intensive Care Department, University Hospital Virgen del Rocio, 41013 Seville, Spain; teresaaldabo@gmail.com

**Keywords:** COVID-19, antimicrobial stewardship, anti-infective agents, bacteremia, candidemia

## Abstract

During the COVID-19 pandemic, the implementation of antimicrobial stewardship strategies has been recommended. This study aimed to assess the impact of the COVID-19 pandemic in a tertiary care Spanish hospital with an active ongoing antimicrobial stewardship programme (ASP). For a 20-week period, we weekly assessed antimicrobial consumption, incidence density, and crude death rate per 1000 occupied bed days of candidemia and multidrug-resistant (MDR) bacterial bloodstream infections (BSI). We conducted a segmented regression analysis of time series. Antimicrobial consumption increased +3.5% per week (*p* = 0.016) for six weeks after the national lockdown, followed by a sustained weekly reduction of −6.4% (*p* = 0.001). The global trend for the whole period was stable. The frequency of empirical treatment of patients with COVID-19 was 33.7%. No change in the global trend of incidence of hospital-acquired candidemia and MDR bacterial BSI was observed (+0.5% weekly; *p* = 0.816), nor differences in 14 and 30-day crude death rates (*p* = 0.653 and *p* = 0.732, respectively). Our work provides quantitative data about the pandemic effect on antimicrobial consumption and clinical outcomes in a centre with an active ongoing institutional and education-based ASP. However, assessing the long-term impact of the COVID-19 pandemic on antimicrobial resistance is required.

## 1. Introduction

Since the declaration on 31 December 2019 of a cluster of cases of pneumonia in Wuhan caused by the Severe Acute Respiratory Syndrome Coronavirus 2 (SARS-CoV-2) virus, more than 50 million cases have been diagnosed worldwide with a mortality rate of 2.5% [1]. In Europe, Spain became one of the epicentres of the emergence and subsequent pandemic of the coronavirus disease 2019 (COVID-19) with a rapidly increasing number of cases [2]. This fact led the Spanish government to declare a national lockdown on 14 March.

For the COVID-19 pandemic, major adjustments of both the healthcare system and frameworks have been required to limit the spread of the virus and avoid unintended collateral effects such as inappropriate antimicrobial prescription and its consequences on antimicrobial resistance [3,4,5,6,7].

Recent studies have reported an unjustified increase of antimicrobial use during the COVID-19 pandemic [8], despite recommendations against it unless bacterial or fungal coinfections were demonstrated [9,10]. The impact of the overuse of these therapies on the propagation of antimicrobial resistance could be an indirect adverse consequence of the pandemic [11,12,13].

Antimicrobial stewardship programmes (ASPs) have been recognised as a potential tool to optimise the use of antimicrobial agents in healthcare centres and hospitals, improving patient outcomes, reducing adverse events, as well as the selection pressure associated with antimicrobial use [14]. Throughout the COVID-19 pandemic, the implementation of antimicrobial stewardship strategies, based on the best available evidence, has been recommended [15,16,17,18,19].

In our hospital, an ASP named Institutional Programme for the Optimisation of Antimicrobial Treatment (PRIOAM) began in 2011. Since its implementation, antimicrobial use, incidence, and death rates of multidrug-resistant (MDR) organisms associated with hospital-acquired bloodstream infections (BSI) significantly decreased [20,21].

The objective of this study was to assess the impact of the COVID-19 pandemic on antimicrobial consumption and the incidence and death rates of hospital-acquired BSI caused by MDR organisms in adult patients admitted to a hospital with an active ongoing ASP.

## 2. Results

**Figure 1:**
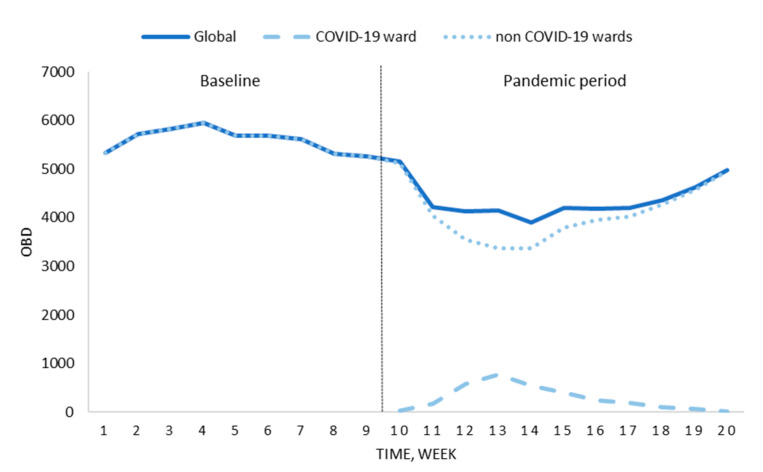
Changes in occupied bed days (OBD) before the national lockdown (baseline) and the pandemic period.

The pandemic period in our centre lasted 11 weeks, from 9 March to 24 May 2020. During this time, 282 patients diagnosed with infection by SARS-CoV-2 were admitted to the hospital, 45 (16.0%) required intensive care, and 55 (19.5%) finally died. As shown in Figure 1, the total number of hospital stays was reduced during this timeframe because of the suspension of scheduled surgical and medical activity (23.1% less scheduled admissions than in the same period in 2019).

On the other hand, reports from the hospital 2019–2020 flu season showed a smaller number of adults admitted with influenza diagnosis compared to the 2018–2019 season (143 vs. 193 patients, respectively).

### 2.1. Antimicrobial Consumption

During the 1st and the 2nd quarter of 2020, antimicrobial consumption increased +99.4 and +53.1 DDD per 1000 OBD, respectively, compared to the same quarters of 2019. Supplementary Materials Figure S1 shows that the reduction in antimicrobial consumption achieved after the implementation of PRIOAM was maintained throughout the following years.

**Figure 2:**
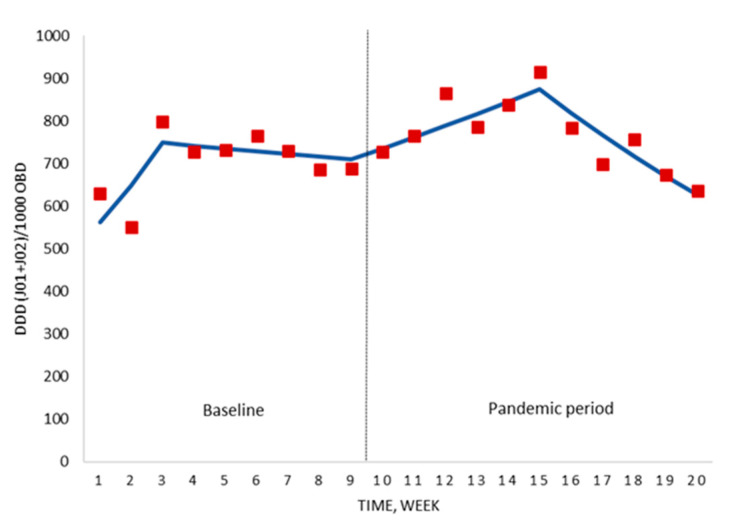
Segmented regression analysis of the hospital antimicrobial consumption for the period before the national lockdown (baseline) and the pandemic period. Red boxes represent the observed value for DDD/1000 OBD, and solid lines are the modelled regression trend segments characterised by the segmented joinpoint regression analysis. DDD, defined daily doses; ATC group J01 (antibacterials for systemic use) and J02 (antifungals); OBD, occupied bed days.

Analysing the results over the course of the COVID-19 pandemic (weeks 10–20), the antimicrobial consumption showed an increase of +3.5% per week (*p* = 0.016) from the beginning until week 15 (six weeks after national lockdown). From this date until the end of the study period, a sustained weekly reduction of −6.4% (*p* = 0.001) was observed (Figure 2; Table S1). The global trend for the whole study period was stable (+0.6% weekly change; *p* = 0.614).

To deduce whether these changes were due to COVID-19, we compared the detailed consumption information for the COVID-19 ward and the hospital without the COVID-19 ward. During the pandemic period (weeks 10–20), no difference was shown regarding antimicrobial prescription (700.3 ± 354.8 vs. 781.8 ± 104.0 DDD per 1000 OBD; *p* = 0.479), even though the antimicrobial use for weeks 16–20 was significantly higher in the COVID-19 ward (Table 1).

**Figure 3:**
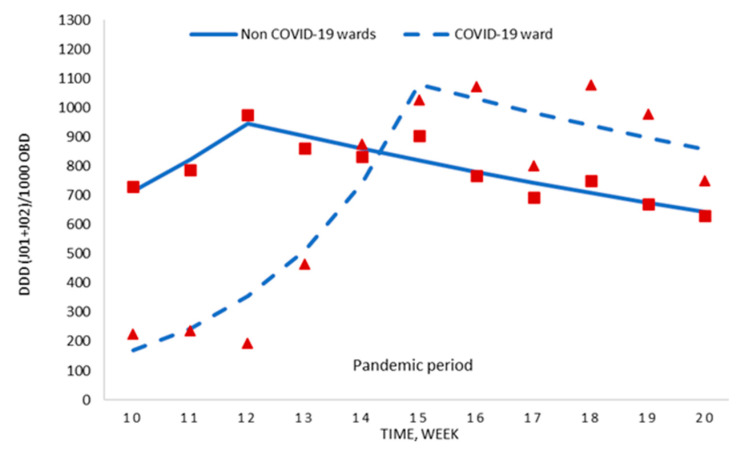
Segmented regression analysis of antimicrobial consumption for the COVID-19 ward and the hospital without the COVID-19 ward during the pandemic period. Red boxes (non COVID-19 wards) and red triangles (COVID-19 ward) represent the observed value for DDD/1000 OBD, and solid lines (non COVID-19 wards) and dashed lines (COVID-19 ward) are the modelled regression trend segments characterised by the joinpoint regression analysis. DDD, defined daily doses; ATC group J01 (antibacterials for systemic use) and J02 (antifungals); OBD, occupied bed days.

The segmented regression analyses of these data are displayed in Figure 3 and Table S2. For the first six weeks of the pandemic period, the weekly antimicrobial use increased in the non COVID-19 wards as well as the COVID-19 ward, with the latter increase being statistically significant (+45.0% weekly change; *p* = 0.006). Thereafter, the consumption in both areas decreased, mainly in the non COVID-19 wards (−4.7% weekly change; *p* = 0.003).

The frequency of empirical antibacterial treatment of patients with SARS-CoV-2 infection and pneumonia at hospital admission was 33.7% (95/282 patients).

### 2.2. Incidence of Hospital-Acquired Candidemia and MDR Bacterial BSI

In the 1st quarter of 2020, the incidence of candidemia and MDR bacterial BSI was 0.37 cases per 1000 OBD vs. 0.23 cases per 1000 OBD in the 1st quarter of 2019. In the 2nd quarter of 2020, it was 0.24 cases per 1000 OBD vs. 0.31 cases per 1000 OBD in the 2nd quarter of 2019. The incidence density of hospital-acquired candidemia and MDR bacterial BSI since the inception of PRIOAM is shown in Figure S2.

For the 20 weeks that comprised the study period, the mean incidence density of BSI caused by MDR organisms remained stable, with a value of 0.36 ± 0.42 cases per 1000 OBD in the COVID-19 period and 0.33 ± 0.28 BSI per 1000 OBD in the period before the national lockdown (*p* = 0.890; see Table 2).

No change in the global trend of incidence of hospital-acquired candidemia and MDR bacterial BSI was detected in the segmented regression analysis (Figure S3; Table S3).

### 2.3. Death Rate of Hospital-Acquired Candidemia and MDR Bacterial BSI

The death rate on day +14 of hospital-acquired candidemia and MDR bacterial BSI in the 1st quarter of 2020 was 0.07 cases per 1000 OBD vs. 0.04 cases per 1000 OBD in the 1st quarter of 2019. In the 2nd quarter of 2020, it was 0.05 cases per 1000 OBD vs. 0.07 cases per 1000 OBD in the 2nd quarter of 2019. The death rates on day +30 were 0.10 and 0.04 cases per 1000 OBD in the 1st quarter of 2020 and 2019, respectively, and it was the same in the 2nd quarters of both 2020 and 2019 (0.07 cases per 1000 OBD). Crude death rates of MDR organisms are represented in Figures S4 and S5.

Table 2 reports the mean values of crude death rates by MDR agents. No differences were observed between the period before the national lockdown and the pandemic period. During these weeks, 17.6% and 26.5% of patients with hospital-acquired BSI caused by MDR organisms died on days +14 and +30, respectively.

## 3. Discussion

During the SARS-CoV-2 virus pandemic, an increase in hospital antimicrobial consumption was observed from inception to six weeks after the national lockdown declaration. This trend was reverted from this point onwards, reaching initial antimicrobial prescription values at the end of the study.

The rise in antimicrobial use occurred in both the COVID-19 ward and the non COVID-19 wards. However, at the time of writing, no studies have communicated the effect of the pandemic outside the COVID-19 ward framework.

For the COVID-19 ward, several studies have reported an increment in antimicrobial prescriptions. In a preliminary analysis of the available data for 2020, the Spanish Strategic Action Plan to reduce the risk of selection and dissemination of antibiotic resistance (PRAN) confirmed a significant increase in antibiotic use for the hospital setting and a notable drop in the community care during March and April due to the pandemic [22]. In another study published by researchers from the Bellvitge University Hospital (Catalonia, Spain) [23], the COVID-19 pandemic significantly increased the overall monthly antibiotic usage compared to the previous year through March and April 2020 with a biphasic pattern: a first wave of empirical antibiotic therapy and a second one with higher use of broad-spectrum antibiotics. In Asia, data from the Singapore General Hospital, with an existing local ASP, showed a 25.5% increase in the prescription of broad-spectrum antibiotics and an increment in the average use of antibiotics in community-onset pneumonia (+2.07 DDD per 100 bed days) and the mean proportion of patients treated with antibiotics (+2.5%) during the pandemic compared to the same period in 2019 [24].

According to our results, the antimicrobial consumption in the COVID-19 ward was low and, despite the increasing trend, the values of DDD per 1000 OBD remained within the range of usual hospital consumption during the first phase of the pandemic. This finding supports the fact that the frequency of empirical treatment of patients diagnosed with SARS-CoV-2 virus infection in our centre was lower compared to other studies. Lai et al. [25] reported that empirical antibiotics were prescribed for 90% of patients in spite of the low confirmation of secondary bacterial infections (10%). Wei et al. [26] also noted that antibiotic prescriptions were initiated on admission in 59% of COVID-19 patients, 98% empirically. Similarly, data on COVID-19 cases, mostly from Asia, reported that while 70% of patients received antimicrobial treatment, only 10% had either bacterial or fungal coinfections [8]. Finally, a recent review and meta-analysis that included 28 primary studies and 3448 patients with COVID-19, concluded that the proportion of COVID-19 patients with bacterial infection was 7.1% (95% CI 4.6–9.6%) and 71.3% of patients received antibiotics (95% CI 57.1–85.5%) [27]. In our case, the low rate of empirical treatment reflects good compliance with the local clinical guidelines (available at http://guiaprioam.com/).

Nevertheless, during the last five weeks of the period, the COVID-19 ward experienced high consumption values compared to those observed in the previous weeks. This fact could be explained by the presence of patients with severe disease, long hospital stays, and hospital-acquired infections. This phenomenon has been proposed by other authors in previous studies [23,28].

The abuse of antimicrobials in acute care settings can stimulate the development of MDR strains [7,11,12,13]. In this sense, a retrospective observational study analysed the bimonthly incidence of carbapenem-resistant *Enterobacteriaceae* (CRE) colonisation and the incidence of CRE acquisition in an Italian intensive care unit from January 2019 to June 2020 and reported that the incidence of CRE acquisition went from 6.7% in 2019 to 50% in March–April 2020. Authors stated that the high intensity of care, the need to be changed in a prone position and prolonged contact with the patient, and the presence of health personnel without work experience in intensive care setting contributed to the spread of CRE acquisition [29]. In our centre, the incidence and death rates of hospital-acquired candidemia and MDR bacterial BSI showed a stable trend and, moreover, the quality of care measured by the percentage of deaths in patients diagnosed with BSI did not deteriorate. Notwithstanding, assessing the long-term impact of the COVID-19 pandemic on antimicrobial resistance is required.

For all the above, we hypothesise that the PRIOAM, its institutional support, and the incorporation of its measures by the prescribers, may have contributed to maintain these results during this extraordinarily difficult situation. Further studies are needed related to the impact of the COVID-19 pandemic in hospitals where ASP had been implemented in the routine clinical practice. To the best of our knowledge, this is the first work specifically focused on providing quantitative data about the pandemic effect on antimicrobial consumption and clinical outcomes in a centre with an active ongoing institutional and education-based ASP.

However, this study has some limitations that must be mentioned. First, as a single-centre study, there may be local factors that preclude extrapolation to other centres. For instance, the disease prevalence, smaller than other regions in Spain and around the world, potentially limits external validity. Secondly, detailed data on specific antimicrobials used, such as ATC groups prescribed, were not included. In addition, data related to antimicrobial consumption in COVID-19 patients admitted to intensive care units and those in conventional hospitalisation could not be disaggregated with accuracy. Thirdly, because of the difficulty of obtaining weekly data retrospectively, it was not possible to compare the 2020 data with those of the previous year. Finally, because we tried to assess the ecological impact of the COVID-19 pandemic on stewardship indicators recorded routinely since PRIOAM inception in 2011 until now, detailed information about all inpatients before and during the pandemic period was not registered.

## 4. Materials and Methods

### 4.1. Setting

This study was performed at the University Hospital Virgen del Rocio (Seville, Spain), a teaching hospital providing a tertiary-care service with 1113 beds (including 62 adult intensive care unit beds), with active solid-organ and haematopoietic stem-cell transplantations programmes. Paediatric wards (181 beds in total) were excluded from the analysis.

### 4.2. Study Design and Period

We conducted a quasi-experimental before-after study of interrupted time-series. Data were prospectively recorded since the inception of the PRIOAM in January 2011 until June 2020, spanning 38 quarters.

To assess in detail the effect of COVID-19 on the PRIOAM outcomes, we conducted a segmented regression analysis for the period before the national lockdown (2nd week of January to 1st week of March: 9 weeks) and the pandemic period (2nd week of March—coinciding with the official national lockdown declaration—to 3rd week of May: 11 weeks).

### 4.3. Hospital Response for the COVID-19 Pandemic

PRIOAM has remained continuously operational throughout the entire hospital since January 2011. The methodology and general results have already been published [20,21].

During the COVID-19 pandemic period, the level of implementation of antimicrobial stewardship strategies was significantly reduced. Instead, most efforts were focused on limiting the spread of the SARS-CoV-2 and on managing patients diagnosed with COVID-19. This situation required the deployment of several adjustments in the hospital organisation and, specifically in the Infectious Diseases Department, such as (a) establishment of multidisciplinary teams led by Infectious Diseases physicians to attend COVID-19 patients; (b) differentiation of a “clean area” for SARS-CoV-2 uninfected patients and a “contaminated area” for patients diagnosed with COVID-19 in the emergency rooms, conventional hospitalisation, and intensive care units; (c) periodical training sessions on the use of personal protective equipment; (d) daily online meetings about local pandemic evolution and patient’s management; (e) weekly online hospital clinical meetings concerning the COVID-19 pandemic; (f) development of local clinical guidelines for the treatment of SARS-CoV-2 infection, available at http://guiaprioam.com/, in which it is specifically recommended not to prescribe empirical antibiotics unless there is clinical suspicion of a bacterial infection.

### 4.4. Study Measures

Antimicrobial consumption was evaluated through weekly measurements of the prescription of antibacterials for systemic use (ATC group J01) and antifungals (ATC group J02). Consumption was calculated as defined daily doses (DDD) per 1000 occupied bed days (OBD), according to the Anatomical Therapeutical Chemical Classification (ATC) methodology and the WHO DDD values [30].

For the same period, most frequent causal agents of hospital-acquired BSI were recorded weekly: *Escherichia coli*, *Klebsiella pneumoniae*, *Acinetobacter baumannii*, *Pseudomonas aeruginosa*, *Staphylococcus aureus,* and *Candida* spp. All-cause crude death rate (all-cause deaths per 1000 OBD) on day +14 and +30 after the diagnosis of an episode of hospital-acquired BSI was also registered weekly.

The quarterly results of these measures were also recorded to analyse the impact of PRIOAM throughout time.

Additionally, hospital admissions due to seasonal influenza epidemic in 2018–2019 and 2019–2020 were assessed in order to control potential confounding variables.

### 4.5. Microbiological Information

Hospital-acquired BSI were those diagnosed by blood cultures obtained > 48 h after admission.

The study of antibiotic susceptibility and resistance was based on EUCAST criteria [31,32]. The MDR categorisation fulfilled the German Society for Hygiene and Microbiology criteria for MDR organisms [33].

### 4.6. Statistical Analysis

Descriptive statistics of the variables were included: categorical variables were presented as frequency distribution and percentages, and continuous variables were presented as means ± standard deviations (SD). Univariate pre-post analyses were performed using Student’s *t*-test and Mann–Whitney U test, after checking for normality using the Kolmogorov–Smirnov test.

We used a segmented joinpoint regression [34] to model trends over time and identify any statistically significant changes that occurred in the linear slope of the trend for each variable using the Joinpoint Regression Program [35], which takes the data (in this case, values for DDD/1000 OBD) and fits the simplest significant joinpoint model that the time-series data allow. The software also enables testing whether an apparent change in trend is statistically significant using a Monte Carlo Permutation method [34]. Joinpoint regression analysis has been identified as a valuable tool for making inferences about changes in trends over time in previous ecological studies [36,37,38].

Confidence intervals (95% CI) or *p*-values were calculated to show statistical significance. Differences were considered statistically significant at *p* < 0.05 (2-tailed tests). Statistical analyses were conducted with IBM SPSS Statistics software, version 23.0 and Joinpoint Regression Program [35] v. 4.7.0.0.

### 4.7. Ethics Approval

The study was conducted in accordance with the Declaration of Helsinki, and the protocol was approved by the Ethics Committee of the University Hospital Virgen del Rocio (Project identification code PI-0361-2010).

## 5. Conclusions

The results of our study demonstrate the effect of the COVID-19 pandemic on antimicrobial consumption and the incidence and death rates of hospital-acquired candidemia and MDR bacterial BSI in adult patients admitted to a tertiary-care hospital with an ASP based on educative measures. The outcomes show that the initial trend of higher antimicrobial use, reached during the first weeks after the national lockdown declaration, was subsequently reverted to a downward trend until the end of the period. Clinical impact, regarding the incidence and death rate of hospital-acquired candidemia and MDR bacterial BSI, was not observed. The PRIOAM may have contributed to maintain these results during the COVID-19 pandemic.

## Figures and Tables

**Table 1:** Antimicrobial consumption for the COVID-19 ward and the hospital without the COVID-19 ward during the pandemic period (weeks 10–20).

Time, Weeks	COVID-19 Ward	Non COVID-19 Wards	Difference of Means (95% CI)	*p*-Value
10–15	503.8 ± 362.3	848.5 ± 86.0	344.6 (−34.5–723.8)	0.067
16–20	936.0 ± 152.5	701.9 ± 56.2	−234.1 (−420.1–−48.0)	0.023
10–20	700.3 ± 354.8	781.8 ± 104.0	81.6 (−162.0–325.1)	0.479

Data are presented as mean ± standard deviation of weekly defined daily doses (DDD) per 1000 occupied bed days (OBD). CI = confidence interval.

**Table 2:** Change in the incidence density and crude death rate of hospital-acquired bloodstream infections (BSI) between the period before the national lockdown (baseline) and the COVID-19 pandemic period.

Outcomes	Baseline	COVID-19 Period	*p*-Value
ID MDR	0.33 ± 0.28	0.36 ± 0.42	0.890
ID total	1.06 ± 0.49	1.10 ± 0.62	0.827
CDR on day +14 MDR	0.06 ± 0.09	0.07 ± 0.16	0.653
CDR on day +14 total	0.16 ± 0.14	0.13 ± 0.20	0.570
CDR on day +30 MDR	0.08 ± 0.09	0.11 ± 0.17	0.732
CDR on day +30 total	0.24 ± 0.17	0.26 ± 0.23	0.789

Data are presented as mean ± standard deviation of weekly incidence density (ID) and crude death rate (CDR). MDR = multidrug-resistant organisms.

## Supplementary Materials

The following are available online at https://www.mdpi.com/2079-6382/9/11/816/s1, Figure S1: Changes in antibiotic consumption since PRIOAM implementation, Figure S2: Changes in the incidence of hospital-acquired bloodstream infections (BSI) produced by multidrug-resistant (MDR) organisms since PRIOAM implementation, Figure S3: Segmented regression analysis of hospital-acquired candidemia and multidrug-resistant (MDR) bacterial bloodstream infections (BSI) for the period before the national lockdown (baseline) and the pandemic period, Figure S4: Changes in the cases of deaths per 1000 occupied bed days (OBD) on day +14 of hospital-acquired bloodstream infections (BSI) produced by multidrug-resistant (MDR) organisms since PRIOAM implementation, and Figure S5: Changes in the cases of deaths per 1000 occupied bed days (OBD) on day +30 of hospital-acquired bloodstream infections (BSI) produced by multidrug-resistant (MDR) organisms since PRIOAM implementation, Table S1: Segmented regression analysis of antimicrobials consumption for the period before the national lockdown and the pandemic period, Table S2: Segmented regression analysis of antimicrobials consumption for the COVID-19 ward and the hospital without the COVID-19 ward during the pandemic period (weeks 10–20), Table S3: Segmented regression analysis of hospital-acquired candidemia and multidrug-resistant (MDR) bacterial bloodstream infections (BSI) for the period before the national lockdown and the pandemic period.
