# Supplementary material for: Impact of the COVID-19 Pandemic on Antimicrobial Consumption and Hospital-Acquired Candidemia and Multidrug-Resistant Bloodstream Infections
Source: Antibiotics (Basel). 2020 Nov 17;9(11):816. doi: 10.3390/antibiotics9110816 (PMC7698104; doi:10.3390/antibiotics9110816)
Supplement: Supplementary file 1 [file antibiotics-09-00816-s001.pdf]

# Supplementary Materials

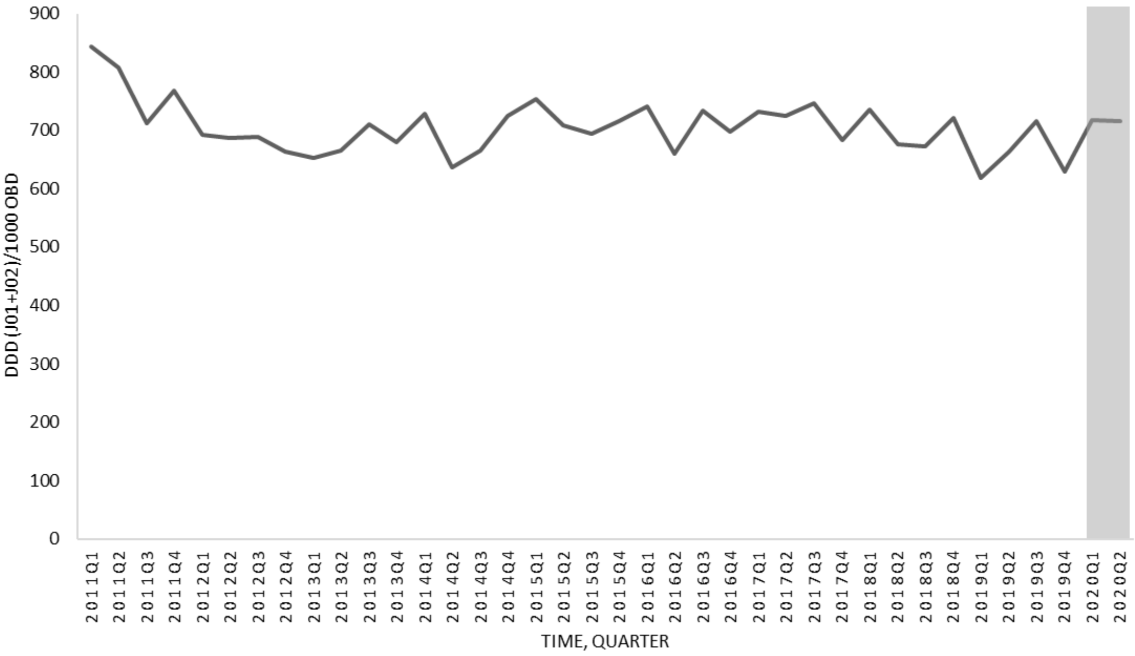


**Figure S1.** Changes in antibiotic consumption since PRIOAM implementation. DDD, defined daily doses; ATC group J01 (antibacterials for systemic use) and J02 (antifungals); OBD, occupied bed days; Q, quarter.

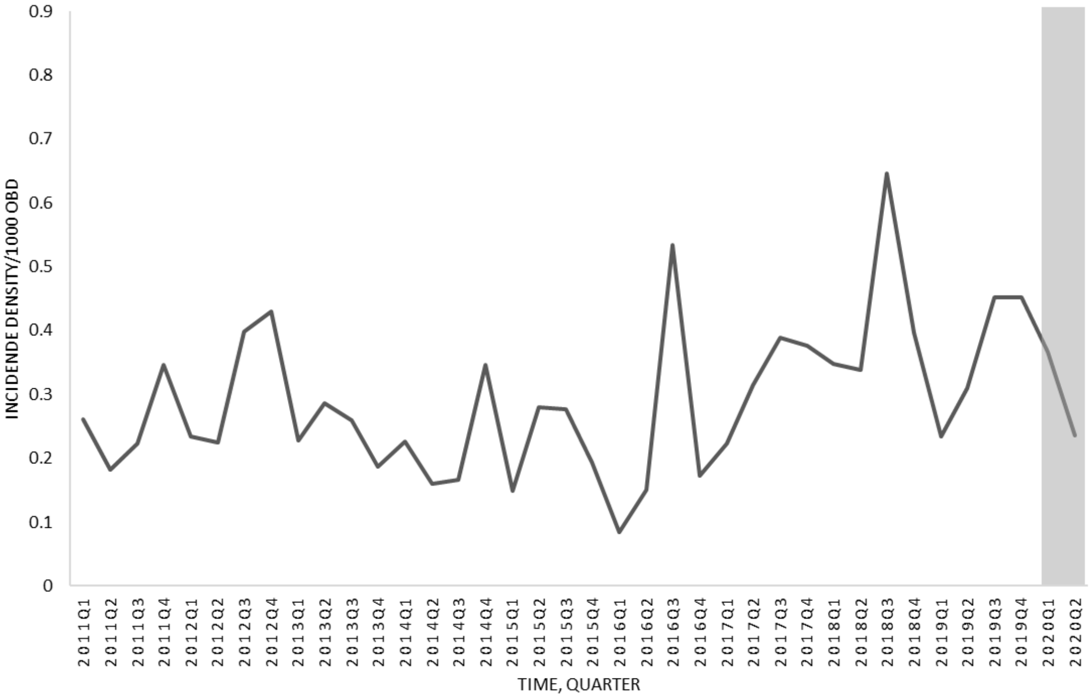


**Figure S2.** Changes in the incidence of hospital-acquired bloodstream infections (BSI) produced by multidrug-resistant (MDR) organisms since PRIOAM implementation. OBD, occupied bed days; Q, quarter.

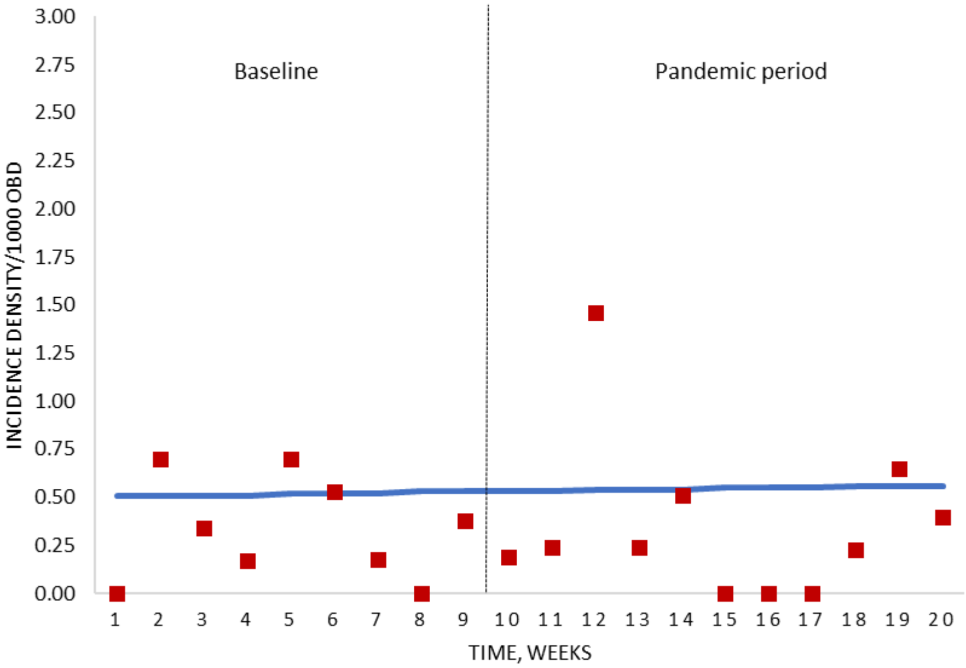


**Figure S3.** Segmented regression analysis of hospital-acquired candidemia and multidrug-resistant (MDR) bacterial bloodstream infections (BSI) for the period before the national lockdown (baseline) and the pandemic period. OBD, occupied bed days.

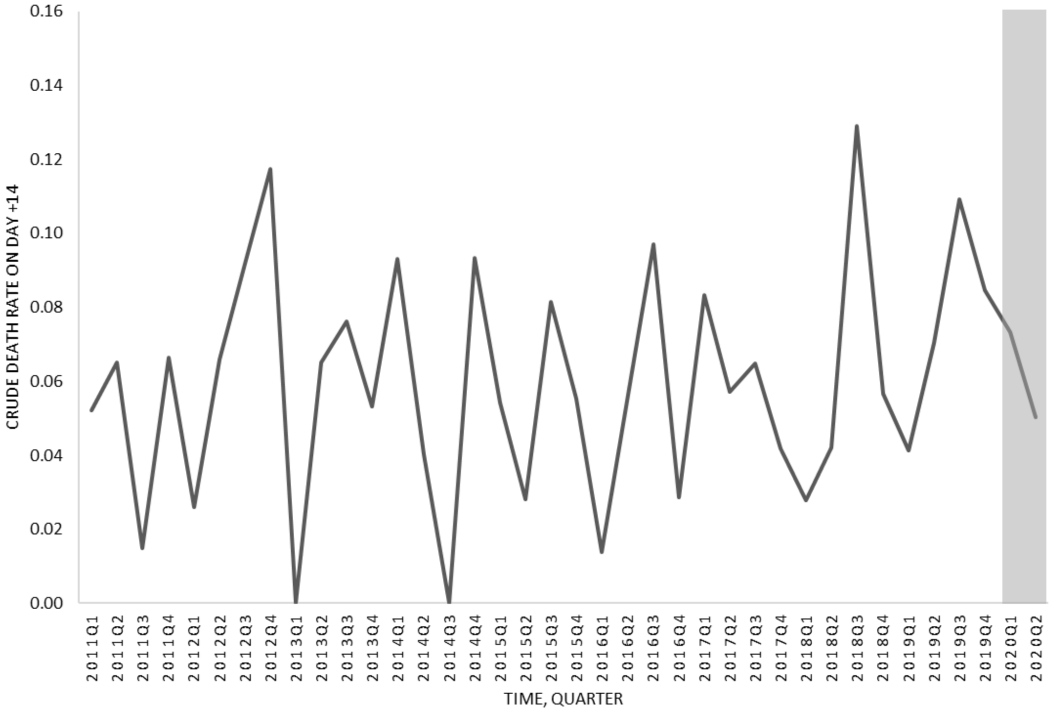


**Figure S4.** Changes in the cases of deaths per 1000 occupied bed days (OBD) on day +14 of hospital-acquired bloodstream infections (BSI) produced by multidrug-resistant (MDR) organisms since PRIOAM implementation. Q, quarter.

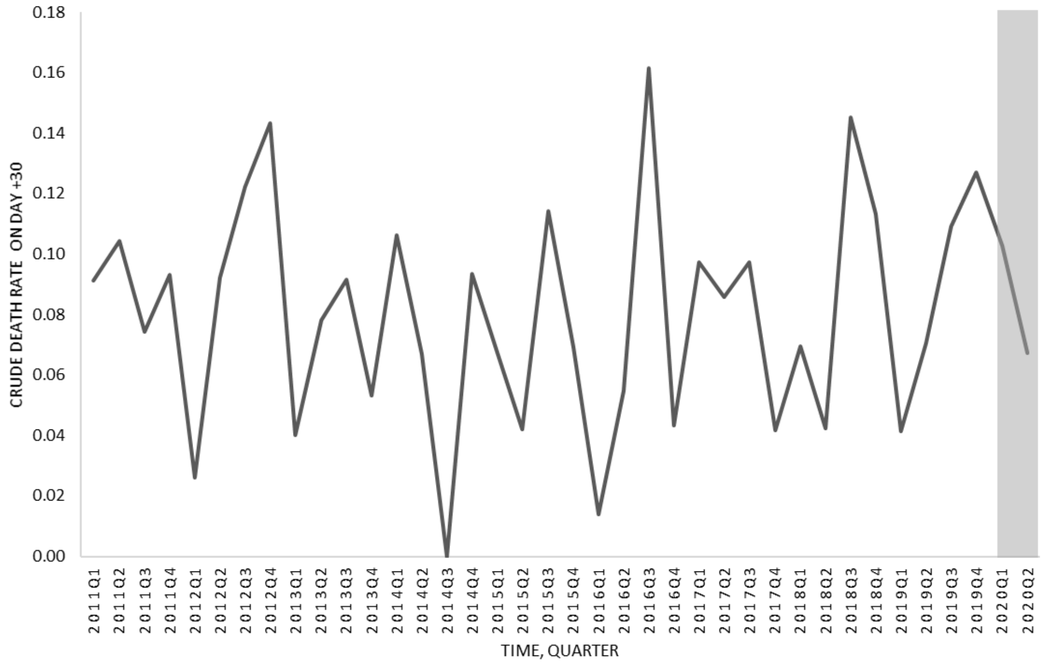


**Figure S5.** Changes in the cases of deaths per 1000 occupied bed days (OBD) on day +30 of hospital-acquired bloodstream infections (BSI) produced by multidrug-resistant (MDR) organisms since PRIOAM implementation. Q, quarter.

**Table S1.** Segmented regression analysis of antimicrobials consumption for the period before the national lockdown and the pandemic period.

| Time, Weeks | WPC | 95% CI | *p*-value |
|---|---|---|---|
| **1 to 3** | 15.471 | -6.389 to 42.436 | 0.155 |
| **3 to 9** | -0.863 | -3.573 to 1.924 | 0.497 |
| **9 to 15** | 3.530 | 0.822 to 6.310 | 0.016 |
| **15 to 20** | -6.434 | -9.080 to -3.711 | 0.001 |
| **Full range** | 0.588 | -1.678 to 2.906 | 0.614 |

Data are presented as weekly defined daily doses (DDD) per 1000 occupied bed days (OBD). WPC=weekly percentage change. CI=confidence interval.

**Table S2.** Segmented regression analysis of antimicrobials consumption for the COVID-19 ward and the hospital without the COVID-19 ward during the pandemic period (weeks 10-20).

| Description | Time, Weeks | WPC | 95% CI | *p*-value |
|---|---|---|---|---|
| **Non COVID-19 wards** | 10 to 12 | 15.027 | -7.285 to 42.708 | 0.163 |
| | 12 to 20 | -4.707 | -6.940 to -2.420 | 0.003 |
| | Full range | -1.051 | -4.716 to 2.754 | 0.583 |
| **COVID-19 ward** | 10 to 15 | 44.951 | 16.382 to 80.532 | 0.006 |
| | 15 to 20 | -4.498 | -17.387 to 10.402 | 0.467 |
| | Full range | 17.657 | 5.891 to 30.730 | 0.002 |

Data are presented as weekly defined daily doses (DDD) per 1000 occupied bed days (OBD). WPC=weekly percentage change. CI=confidence interval.

**Table S3.** Segmented regression analysis of hospital-acquired candidemia and multidrug-resistant (MDR) bacterial bloodstream infections (BSI) for the period before the national lockdown and the pandemic period.

| Time, Weeks | WPC | 95% CI | *p*-value |
|---|---|---|---|
| **Full range** | 0.541 | -4.164 to 5.477 | 0.816 |

Data are presented as weekly cases per 1000 occupied bed days (OBD). WPC=weekly percentage change. CI=confidence interval.
